# Aluminum overload increases oxidative stress in four functional brain areas of neonatal rats

**DOI:** 10.1186/1423-0127-19-51

**Published:** 2012-05-21

**Authors:** Chia-Yi Yuan, Yih-Jing Lee, Guoo-Shyng Wang Hsu

**Affiliations:** 1 Graduate Institute of Nutrition and Food Sciences, Fu-Jen Catholic University, Hsinchuang, New Taipei City, Taiwan; 2 School of Medicine, Fu-Jen Catholic University, 510 Chung-Cheng Road, Hsinchuang, New Taipei City, 24205, Taiwan; 3 Department of Nutritional Science, Fu-Jen Catholic University, 510 Chung-Cheng Road, Hsinchuang, New Taipei City, 24205, Taiwan

**Keywords:** Aluminum, Neonatal rats, Functional brain tissues, Intraperitoneal injection

## Abstract

**Background:**

Higher aluminum (Al) content in infant formula and its effects on neonatal brain development are a cause for concern. This study aimed to evaluate the distribution and concentration of Al in neonatal rat brain following Al treatment, and oxidative stress in brain tissues induced by Al overload.

**Methods:**

Postnatal day 3 (PND 3) rat pups (n =46) received intraperitoneal injection of aluminum chloride (AlCl_3_), at dosages of 0, 7, and 35 mg/kg body wt (control, low Al (LA), and high Al (HA), respectively), over 14 d.

**Results:**

Aluminum concentrations were significantly higher in the hippocampus (751.0 ± 225.8 ng/g v.s. 294.9 ± 180.8 ng/g; *p* < 0.05), diencephalon (79.6 ± 20.7 ng/g v.s. 20.4 ± 9.6 ng/g; *p* < 0.05), and cerebellum (144.8 ± 36.2 ng/g v.s. 83.1 ± 15.2 ng/g; *p* < 0.05) in the HA group compared to the control. The hippocampus, diencephalon, cerebellum, and brain stem of HA animals displayed significantly higher levels of lipid peroxidative products (TBARS) than the same regions in the controls. However, the average superoxide dismutase (SOD) activities in the cerebral cortex, hippocampus, cerebellum, and brain stem were lower in the HA group compared to the control. The HA animals demonstrated increased catalase activity in the diencephalon, and increased glutathione peroxidase (GPx) activity in the cerebral cortex, hippocampus, cerebellum, and brain stem, compared to controls.

**Conclusion:**

Aluminum overload increases oxidative stress (H_2_O_2_) in the hippocampus, diencephalon, cerebellum, and brain stem in neonatal rats.

## Background

Increasing evidence has demonstrated that oxidative stress is the primary cause of pathogenesis in inflammatory, partial ischemia, metabolic, and denatured cranial nerve disease [1]. Brain tissues are highly susceptible to oxidative damage, probably because of high oxygen consumption rate (20%), the presence of abundant polyunsaturated fatty acids in cell membranes, high iron (Fe) content, and low anti-oxidative enzyme activities [2]. Although aluminum (Al) is a relatively low redox mineral, it can induce oxidative damage through multiple mechanisms. It can bind to negatively charged brain phospholipids, which contain polyunsaturated fatty acids and are easily attacked by reactive oxygen species (ROS) such as O_2_^˙-^, H_2_O_2_, OH^˙^, and OH^-^[3]. Aluminum can also stimulate iron-initiated lipid peroxidation in the Fenton reaction, which causes ROS production and Fe^3+^ formation. Superoxide (O_2_^˙-^) is neutralized by Al^3+^ to form an Al-O_2_^˙-^ complex, which increases the oxidative capacity of O_2_^˙-^[4].

Reactive oxygen species may also cause cellular damage, by oxidizing amino acid residues on proteins, forming protein carbonyls [5]. The study of Kowalczyk et al. showed that three months’ administration of aluminum chloride in drinking water at a dose of 80 mg/l significantly elevated protein carbonyl groups concentration in erythrocytes hemolysate [6]. Lesions existed in the brains of Alzheimer’s disease (AD) patients are typically associated with attacks by free radicals, and Al is shown to have catalytic activity to produce free radicals. In addition, beta-amyloid protein in the brain of AD patients is aggregated and induces more free radicals production [7].

Several studies have shown that oxidative stress induced by Al modifies the peroxidation of lipids and the activities of anti-oxidative enzymes. Julka and Gill (1996) [8] identified increased peroxidation of lipids in brain tissues of adult Wistar rats following administration of 10 mg/d Al (aluminum lactate) for 4 wk. Oral administration of Al acetate (4000 mg Al /kg body wt and 6000 mg Al /kg body wt) for 8 wk resulted in higher lipid peroxidative products (thiobarbituric acid reactive substances, or TBARS) in mouse brain tissue lysates [9]. In the study of Katyal et al, aluminum treatment increased hyperoxidation of cerebral proteins [10]. A different study, however, observed that lower Al^3+^ (12.5 μmol/L) inhibited oxidation of mouse cerebellar tissue proteins, while higher Al^3+^ (25 μmol/L and 50 μmol/L) increased oxidation of the proteins [11]. Aluminum can, therefore, induce protective effects against oxidative stress and also cause damaging effects through oxidative stress.

The main route of Al excretion is the urine; therefore, subjects with kidney malfunction or immature kidney, such as nephropathy patients or neonates, might experience toxic accumulation of Al in the body [12]. Infant formula is the primary food source for bottle-fed neonates. The study of Yuan et al reviewed several other studies and reported that most commercial infant formulas contained higher Al (6.5 μM to 87 μM) than human breast milk (0.2 μM to 1.7 μM) [12]. Infants display rapid growth and their brain-blood-barrier, detoxification system (liver), and excretory system (kidney) are not well-developed [13,14]. Aluminum can cross the blood-brain barrier and accumulate in glial and neural cells [15]. Thus, high intake of Al-containing formula might cause accumulation of Al in the neonatal brain, interfering with appropriate development.

In previous studies, exposure to excess dietary Al during gestation and lactation periods had no toxic effects on the mother, but resulted in persistent neurobehavioral deficits in the pups, such as defects in the sensory motor reflexes, locomotor activity, learning capability, and cognitive behavior [16,17]. These behavioral studies, therefore, suggested that Al exposure might cause developmental changes in neonatal brain. Until recently, a marker with which to effectively detect neonatal brain development was lacking. The group’s previous study with Al treatment in neonatal rat hippocampal neurons at concentrations of 37 μM and 74 μM for 14 days significantly reduced NMDAR (N-methyl-D-aspartate receptor) expression which was used as a marker of brain development. This suggested that Al exposure might influence the development of hippocampal neurons in neonatal rats [12].

In previous studies on adult animals, Al induced the production of ROS and caused oxidative damage in the brain [3-11]. Neonatal animal studies also identified that Al exposure during gestation and lactation periods induced brain developmental deficits in pups [12,16,17]. Thus, the hypothesis of the present study was that ROS might be the cause of developmental damage in the neonatal brain following Al administration. In order to effectively induce an Al overload state in animals, the experiments, therefore, involved intraperitoneal injection of equivalent amounts of Al (AlCl_3_) into PND 3 neonatal rats during a 14 d lactation period. The deposition of Al, oxidative stress related enzyme activities as well as thiobarbituric acid reactive substances (TBARS) in functional areas of growing brain tissue were analyzed.

## Methods

### Experimental design

Postnatal day 3 (PND 3) SD rats were randomly divided into 3 experimental groups, which received intraperitoneal injection of different AlCl_3_ (Merck 801081) loads for 14 d: control (normal saline), low Al (LA, 7 mg Al/kg/d, 1/530 LD_50_), and high Al (HA, 35 mg Al/kg/d, 1/106 LD_50_). The Al content, levels of thiobarbituric acid reactive substances (TBARS), and activities of superoxide dismutase (SOD), catalase, and glutathione peroxidase (GPx) in the cerebral cortex, hippocampus, diencephalon, cerebellum, brain stem, pituitary, and olfactory bulb were evaluated.

### Animals and breeding

Mature Sprague-Dawley (SD) rats were purchased from the Laboratory Animal Center of National Applied Research Laboratories Taiwan. The processes of protocols using experimental animals were approved by the Institutional Animal Care and Use Committee (IACUC) of Fu-Jen University. All rats were kept individually in stainless steel cages under a 12/12 h light–dark cycle, at 20°C to 22°C in a 60% humidity animal room. Standard laboratory chow (Purine Lab. Chow 5001, St. Louis, MO) and DI water (Millipore, Milli-Q Ultra pure water system, 18.2 ΩM/cm) were provided ad lib. After 1 wk adaptation, one male and one female rat were put into the same cage for mating. The pregnant female rats were kept individually in plastic cages for approximately 21 d gestation. Within 24 h of delivery, 10 newborn pups were placed in a cage with the maternal rat and breast-fed. Forty-six neonatal pups were divided into the 3 experimental groups: control (n = 16), LA (n = 16), and HA (n = 14). Based on the study of Guo et al [18], dosages of 0 (control), 7 (LA), and 35 (HA) mg Al/kg body wt./d (AlCl_3_) were intraperitoneally injected into each neonatal pup for 14 d.

### Sample preparation

Animals were anesthetized and decapitated on PND 17. The cerebral cortex, hippocampus, diencephalon, cerebellum, brain stem, pituitary, and olfactory bulb were collected. After weighing, a section of each tissue sample was frozen at -70°C for Al content analysis; the other parts were homogenized on ice using various sample buffers. After centrifugation, supernatants were collected and frozen at -70°C for oxidative product and anti-oxidative enzyme analyses.

### Determination of Al content

After wet acid digestion of brain tissues, the Al contents in all samples were determined by using a flameless atomic absorption spectrophotometer (AAS, 903GBC, GBC, Australia) with a graphite furnace (System 3000, GBC, Australia). The instrument was adjusted at a wavelength of 309.3 nm, with a slit of 0.5 nm and a Hollow cathode Al lamp (GBC). A lamp current of 8.0 mA, integration time of 1 s, double beam, and D_2_ background correction were used.

### Oxidative product and anti-oxidative enzyme assays

The TBARS was measured as the parameter of the lipid peroxidation of samples according to the protocols described previously [19]. Lipid peroxides in the sample by repeat heating with acid would generate the second product which would react with thiobarbituric acid (TBA) to form TBARS with the maximal absorbance in 532 nm.

The anti-oxidative enzyme SOD activity was evaluated using the Cayman Chemical Superoxide Dismutase Assay Kit (chemical Item Number 706002). O_2_^˙-^ and H^+^ produced by lipids in the sample react with hypoxanthine catalyzed by xanthine oxidase to generate the products, H_2_O_2_ and O_2_. The enzyme levels increase with decreasing of substrate, i.e. O_2_^**˙-**^[20].

The anti-oxidative enzyme catalase was evaluated using the Cayman Chemical Catalase Assay Kit (chemical Item Number 707002). Methanol reacts with H_2_O_2_ catalyzed by catalase to form formaldehyde. Formaldehyde and Purpald (4-amino-3-hydrazino- 5-mercapto-1,2,4-triazole) produce a purple color in which the absorbance at 540 nm is positively correlated with the enzyme activity [21].

The anti-oxidative enzyme GPx was evaluated using the Cayman Chemical Glutathione Peroxidase Assay Kit (chemical Item Number 703102). Oxidized glutathione (GSSG), produced upon reduction of an organic hydroperoxide by GPx, is recycled to its reduced state by GR and NADPH. The oxidation of NADPH to NADP^+^ is accompanied by a decrease in absorbance at 340 nm. The rate of decrease in the A340 is directly proportional to the GPx activity in the sample [22].

### Determination of protein level

The protein quantization of each brain tissue was determined by Pierce Coomassie® Plus Protein Assay Reagent Kit (chemical Item Number 23236). Basically when Coomassie**®** Dye binds protein in an acidic medium, an immediate shift in absorption maximum occurs from 465 nm to 595 nm with a concomitant color change from brown to blue [23].

### Statistical analysis

Data are expressed as mean ±  SD and were analyzed using Statistical Analysis System (SAS), version 9.2. One-way analysis of variance (ANOVA) was conducted to detect differences in the measurement values among groups, followed by Schaffer’s test. Pearson’s correlation analysis was used to assess the correlation among variables. A *p*-value < 0.05 was considered statistically significant.

## Results

### Effects of Al on animal growth

Results indicated no significant differences in the body weight gain among the 3 groups during the experimental period (*p* > 0.05). However, the cerebellar weights were significantly lower in the Al groups than in the control group (0.14 ± 0.04 (HA), 0.14 ± 0.03 (LA), 0.21 ± 0.05 (control) g; *p* < 0.05) (Table 1). Similarly, the brain stem weights were significantly lower in the HA group than in the control group (0.19 ± 0.05 g v.s. 0.24 ± 0.06 g; *p* < 0.05). The weights of the olfactory bulb were significantly higher in the HA group than in the LA group (0.08 ± 0.04 g v.s. 0.04 ± 0.01 g; *p* < 0.05). The average cerebellar weight to body weight ratio was significantly lower in the HA group than in the control group (0.48 ± 0.18% v.s. 0.71 ± 0.24%; *p* < 0.05), while the average olfactory bulb weight to body weight ratio was significantly higher in the HA group than in the LA group (0.28 ± 0.15% v.s. 0.16 ± 0.05%; *p* < 0.05) (Table 2).

**Table 1:** **Final body weight, body weight gains, the brain tissues weights (g) of neonatal pups after 2-weeks of intraperitoneal administration of AlCl**_**3**_^**1,2,3**^

	Control (n = 16)	LA (n = 16)	HA (n = 14)
	(g)	(g)	(g)
Final body weight	29.9 ± 3.1	28.6 ± 7.7	30.2 ± 5.1
Body weight gains	21.3 ± 1.5	17.9 ± 9.0	20.7 ± 5.2
Whole brain	1.53 ± 0.28	1.29 ± 0.10	1.36 ± 0.14
Cerebral cortex	0.44 ± 0.12	0.37 ± 0.08	0.40 ± 0.09
Hippocampus	0.09 ± 0.03	0.09 ± 0.01	0.10 ± 0.04
Diencephalon	0.44 ± 0.09	0.39 ± 0.06	0.40 ± 0.06
Cerebellum	0.21 ± 0.05 ^a^	0.14 ± 0.03 ^b^	0.14 ± 0.04 ^b^
Brain stem	0.24 ± 0.06 ^a^	0.21 ± 0.03 ^ab^	0.19 ± 0.05 ^b^
Pituitary gland	0.05 ± 0.01	0.04 ± 0.02	0.05 ± 0.02
Olfactory bulb	0.06 ± 0.02 ^ab^	0.04 ± 0.01 ^b^	0.08 ± 0.04 ^a^

^1^ Values are mean ± SD.

^2^ Intraperitoneal (IP) administered AlCl_3_ of 0, 7, 35 mg Al/kg body wt/day for 2 weeks in control, low Al (LA) and high Al (HA) groups respectively.

^3^ Values in the same raw with different ^a, b^ superscripts are significantly different (p < 0.05).

**Table 2:** **Relative brain tissue weight to body weight ratio in neonatal pups after 2-weeks intraperitoneal administration of AlCl**_**3**_^**1,2,3,4**^

	Control (n = 16)	LA (n = 16)	HA (n = 14)
	(%)	(%)	(%)
Whole brain	5.24 ± 1.51	4.76 ± 1.09	4.66 ± 1.12
Cerebral cortex	1.52 ± 0.53	1.36 ± 0.42	1.36 ± 0.42
Hippocampus	0.30 ± 0.13	0.31 ± 0.07	0.35 ± 0.19
Diencephalon	1.50 ± 0.44	1.47 ± 0.45	1.36 ± 0.29
Cerebellum	0.71 ± 0.24 ^a^	0.53 ± 0.16 ^ab^	0.48 ± 0.18 ^b^
Brain stem	0.83 ± 0.26	0.78 ± 0.21	0.64 ± 0.22
Pituitary gland	0.18 ± 0.05	0.16 ± 0.06	0.18 ± 0.06
Olfactory bulb	0.21 ± 0.09 ^ab^	0.16 ± 0.05 ^b^	0.28 ± 0.15 ^a^

^1^ Values are mean ± SD.

^2^ Intraperitoneal (IP) administered AlCl_3_ of 0, 7, 35 mg Al/kg body wt/day for 2 weeks in control, low Al (LA) and high Al (HA) groups respectively.

^3^ Values in the same raw with different ^a, b^ superscripts are significantly different (p < 0.05).

^4^ Relative tissue weight = brain tissue weight (g) / body weight (g) × 100.

### Aluminum content in different brain tissues

After injection of AlCl_3_ for 2 wk, the hippocampus (751.0 ± 225.8 v.s. 294.9 ± 180.8 ng/g; *p* < 0.05), diencephalon (79.6 ± 20.7 v.s. 20.4 ± 9.6 ng/g; *p* < 0.05), and cerebellum (144.8 ± 36.2 v.s. 83.1 ± 15.2 ng/g; *p* < 0.05) of the HA group displayed significantly higher Al contents than those of the control group (Table 3). A similar trend was observed in the brain stem, although there were no significant differences between the Al-treated and control groups (57.4 ± 21.8 (HA), 41.0 ± 13.0 (LA), 42.0 ± 6.1 (control) ng/g; *p* > 0.05). The Al groups displayed no accumulation of Al in the cerebral cortex, pituitary, and olfactory bulb when compared with the control group. Results indicated that the levels of Al in the diencephalon, hippocampus, cerebellum, and brain stem of the HA group were 4.0-, 2.5-, 1.7-, and 1.3-fold respectively, of that in the control group. Aluminum, thus, more easily aggregated in these 4 regions than in other tissues in the neonatal brain.

**Table 3:** **Al contents in the brain tissues of neonatal pups after 2-weeks intraperitoneal administration of AlCl**_**3**_^**1,2,3**^

	Control (n = 8)	LA (n = 8)	HA (n = 7)
Whole brain (ng/g)	165.5 ± 37.9 ^b^	167.4 ± 32.8 ^b^	219.5 ± 43.1 ^a^
Cerebral cortex (ng/g)	60.9 ± 7.5	61.5 ± 26.1	60.4 ± 11.4
Hippocampus (ng/g)	294.9 ± 180.8 ^b^	492.0 ± 236.8 ^ab^	751.0 ± 225.8 ^a^
Diencephalon (ng/g)	20.4 ± 9.6 ^b^	46.9 ± 33.9 ^b^	79.6 ± 20.7 ^a^
Cerebellum (ng/g)	83.1 ± 15.2 ^b^	88.1 ± 21.6 ^b^	144.8 ± 36.2 ^a^
Brain stem (ng/g)	42.0 ± 6.1	41.0 ± 13.0	57.4 ± 21.8
Pituitary gland (mg/g)	3.08 ± 1.42	2.34 ± 0.49	2.42 ± 0.82
Olfactory bulb (ng/g)	173.9 ± 42.2	183.7 ± 36.9	171.2 ± 75.3

^1^ Values are mean ± SD.

^2^ Intraperitoneal (IP) administered AlCl_3_ of 0, 7, 35 mg Al/kg body wt/day for 2 weeks in control, low Al (LA) and high Al (HA) groups respectively.

^3^ Values in the same raw with different ^a, b^ superscripts are significantly different (p < 0.05).

### Oxidative products in different brain tissues

**Figure 1:**
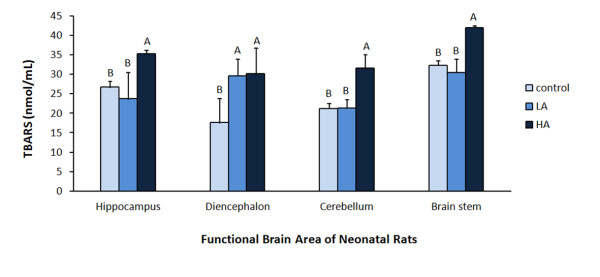
**TBARS concentrations of brain tissues in neonatal pups after 2-weeks intraperitoneal administration of AlCl**_**3.**_ Intraperitoneal (IP) administered AlCl_3_ of 0, 7, 35 mg Al/kg body wt/day for 2 weeks in control, low Al (LA) and high Al (HA) groups respectively. Values in the figure with different ^A, B^ superscripts are significantly different (p < 0.05).

As shown in Figure 1, the hippocampus displayed higher levels of lipid peroxide products in the HA group than in the LA and control groups (35.26 ± 0.88 (HA), 23.67 ± 6.77 (LA), 26.69 ± 1.46 (control) nmol/mL; *p* < 0.05). The diencephalon displayed significantly higher levels of lipid peroxide products in both Al-treated groups than in the control group (30.08 ± 6.71, 29.64 ± 4.26, 17.55 ± 6.31 nmol/mL; *p* < 0.05). Lipid peroxide product levels were significantly higher in the cerebellum in the HA group than in the LA and control groups (31.51 ± 3.48, 21.32 ± 2.23, 21.19 ± 1.33 nmol/mL; *p* < 0.05). Similarly, the brain stem lipid peroxide product levels were significantly higher in the HA group than in the LA and control groups (41.98 ± 0.42, 30.49 ± 3.36, 32.29 ± 1.21 nmol/mL; *p* < 0.05). The levels of lipid peroxidative products (TBARS) in the hippocampus, diencephalon, cerebellum, and brain stem tissues were, thus, significantly higher in the high Al animals compared to the low Al and control animals.

### Anti-oxidative enzyme activities in different brain areas

**Figure 2:**
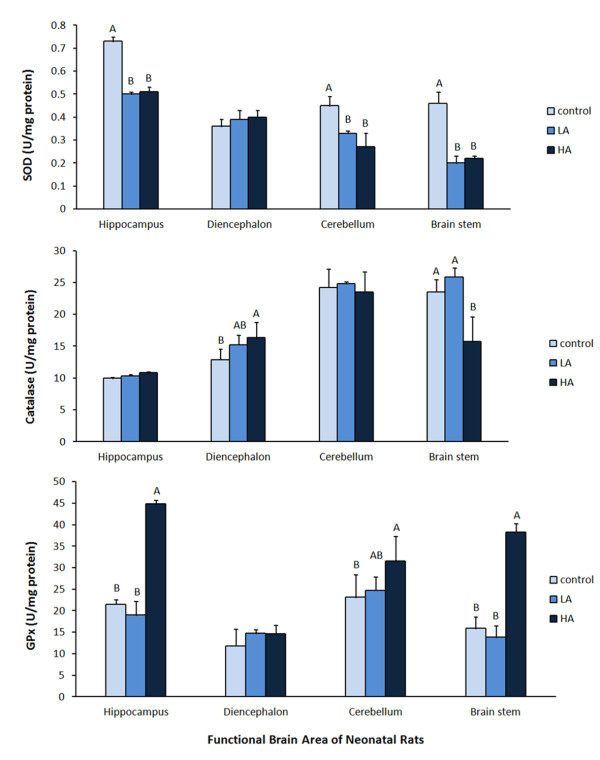
**Specific activities of anti-oxidative enzymes of brain tissues in neonatal pups after 2-weeks intraperitoneal administration of AlCl**_**3.**_ Intraperitoneal (IP) administered AlCl_3_ of 0, 7, 35 mg Al/kg body wt/day for 2 weeks in control, low Al (LA) and high Al (HA) groups respectively. Values in the figure with different ^A, B^ superscripts are significantly different (p < 0.05).

The SOD activities in the hippocampus were significantly lower in the HA (0.51 ± 0.02 unit/mg protein) and the LA (0.50 ± 0.01 unit/mg protein) groups compared to the controls (0.73 ± 0.02 unit/mg protein (Figure 2A)). So were the cerebellum (0.27 ± 0.06, 0.33 ± 0.02, 0.45 ± 0.04 unit/mg protein; *p* < 0.05), and brain stem (0.22 ± 0.02, 0.20 ± 0.03, 0.46 ± 0.05 unit/mg protein; *p* < 0.05).

However, catalase activity in the diencephalon was significantly higher in the HA group than in the control group (16.31 ± 2.45 unit/mg protein v.s. 12.86 ± 1.67 unit/mg protein; *p* < 0.05) (Figure 2B). Conversely, the catalase activity in the brain stem was significantly lower in the HA group than in the LA and control groups (15.78 ± 3.88, 25.90 ± 1.38, 23.52 ± 1.93 unit/mg protein; *p* < 0.05).

The GPx activities in the hippocampus (44.85 ± 0.79, 21.54 ± 1.02, 18.98 ± 3.15 unit/mg protein; *p* < 0.05), cerebellum (31.53 ± 5.79, 23.15 ± 5.29, 24.72 ± 3.16 unit/mg protein; *p* < 0.05), and brain stem (38.29 ± 1.98, 15.91 ± 2.62, 13.81 ± 2.69 unit/mg protein; *p* < 0.05) were significantly higher in the HA group than in the LA and control groups (Figure 2C).

### The correlations among aluminum, oxidative products, and anti-oxidative enzyme activities

The result of correlation analysis indicated that there was a positive correlation between aluminum contents and TBARS in the hippocampus, cerebellum, and brain stem (r = 0.34, *p* = 0.08; r = 0.69, *p* < 0.05; r = 0.45, *p* < 0.05). Meanwhile, there was a positive correlation between aluminum contents and GPx (r = 0.56, *p* < 0.05; r = 0.55, *p* < 0.05; r = 0.49, *p* < 0.05), but a negative correlation existed between aluminum levels and SOD (r = -0.53, *p* < 0.05; r = -0.57, *p* < 0.05; r = -0.18, *p* = 0.37) in the hippocampus, cerebellum, and brain stem. On the other hand, a positive correlation existed between aluminum contents and catalase in the diencephalon (r = 0.56, *p* < 0.05), while there was a negative correlation in the brain stem (r = -0.47, *p* < 0.05).

## Discussion

The initial objective of present study was to evaluate the accumulation of Al in the brains of neonates receiving high Al-containing commercial infant formula. However, it was not possible to conduct the analyses on human newborns. Three-week-old rats were, therefore, used to imitate 6-month-old infants during the breastfeeding period. Although previous studies have suggested that excess Al accumulates in the hippocampus, diencephalon, and cerebellum, no prior investigation has reported Al oxidative damage in various areas of the brain in neonatal rats. The present study’s results suggest that toxic Al concentrations, and oxidative damage caused by Al, occur in the 4 functional areas of the hippocampus, diencephalon, cerebellum, and brain stem in growing brain tissue.

### The pathological toxic concentration of Al in neonatal brain

The results of present study showed that the Al concentration in the whole brain of neonatal rats (PND 17) were 165.5 ng/g, 167.4 ng/g, and 219.5 ng/g which are much lower than the Al concentrations from our previous pilot work, 790 ng/g, 830 ng/g, and 1440 ng/g respectively when neonates were fed artificial rats milk at the dosage of 0, 22.5 and 225 mM AlCl_3_ respectively from PND 3 to 17). In addition, the levels of Al in the diencephalon, hippocampus, and cerebellum, were 1.5–8 fold higher respectively, in the gastrostomy bolus feeding groups (440.0 ± 20.0 (HA) ng/g, 1180.0 ± 30.0 (HA), 1100.0 ± 60.0 (HA) ng/g) than that in the present work (79.6 ± 20.7 (HA) ng/g, 751.0 ± 225.8 (HA), 144.8 ± 36.2 (HA) ng/g). It is suggested that Al levels in neonatal whole brain and/or various functional brain areas through the intraperitoneal injection would not exceed the physiological concentrations of Al from formula gastrostomy bolus feeding.

### The distribution of Al in neonatal rat brain

When evaluating the effects of high Al on brain growth, the results indicated marginal changes in tissue weights in the cerebral cortex, pituitary, and olfactory bulb, as illustrated in Tables 1 and 2. However, no previous study has reported weight changes in these brain areas. Evaluations of Al distribution revealed that Al predominantly accumulated in the hippocampus, diencephalon (including the thalamus, metathalamus, hypothalamus, epithalamus, prethalamus or subthalamus, and pretectum), cerebellum, and brain stem (including the medulla oblongata, pons, and midbrain) of neonatal rats. Previous studies on adult rats have described Al accumulation in the same brain areas. Gomez and colleagues observed high levels of Al in the cortex, hippocampus, and cerebellum [24] of Aβ PP transgenic mice provided 1 mg Al/g feed for 6 mo. Domingo and colleagues further described Al in the cortex, thalamus, and olfactory bulb in rats [25] following administration of Al at doses of 0, 50, and 100 mg Al (Al nitrate)/kg body wt/d in drinking water over 6.5 mo. Sanchez-Iglesias et al have also identified Al accumulation in the cortex, hippocampus, striatum, cerebellum, and ventral midbrain of rats following treatment with intraperitoneal injected or orally drinking with aluminum chloride [26]. The results of the present study indicated that administration of high concentration AlCl_3_ induced higher levels of Al accumulation in the brain stem. Consistent with previous studies on adult rats, distribution of Al mainly occurred in 3 specific areas in neonatal rat brain: the hippocampus, diencephalon, and cerebellum. Higher accumulation of Al also occurred in these brain regions in the neonatal rats. Results indicated limited deposition of Al in the cerebral cortex, pituitary, and olfactory bulb.

### Effects of Al on the production of oxidative products in the hippocampus, diencephalon, cerebellum, and brain stem

The hippocampus, diencephalon, cerebellum, and brain stem tissues of high Al animals displayed significant increases in the levels of lipid peroxidative products (TBARS). Previous research, which involved oral administration of Al (0.1 mmol/kg/d) [27,28] or intraperitoneal injection of Al (7 mg/kg/d) for 11 wk [29], also suggested that high accumulation of Al in the hippocampus increased the lipid peroxidative products. Similarly, Nehru and Bhalla reported elevated Al in the diencephalon of female Sprague Dawley rats administered with AlCl_3_ (40 mg/ kg/d) for 8 wk, accompanied by increased lipid peroxidation in the hypothalamus (part of the diencephalon) [30]. Several other studies have shown that accumulation of Al in the cerebellum increased the lipid peroxidative products. In one study, oral administration of Al (100 mg/kg/d) for 2 mo increased lipid peroxidation in the cerebellum of adult rats [31]. The cerebellar TBARS levels also increased in rats intraperitoneally injected with aluminum lactate (7 mg Al/kg/d) for 11 wk [32]. Nehru et al further identified significant increases in lipid peroxidation in the cerebrum and cerebellum of pup brains following exposure of developed and developing rat brains to oral aluminum chloride (100 mg/kg/d) for 6 wk or 8 wk [33]. Investigations on the brain stem have also demonstrated increased lipid peroxidation in the medulla oblongata [30,34] and ventral midbrain [35] of adult rats. Results, therefore, suggest that increased lipid peroxidation in the brain stem was associated with high Al.

Two animal studies with high level of intraperitoneal injection of Al (7 mg/kg/d) for 11 wk [29], and oral Al intakes 100 mg/kg/d for 2 mo [31], resulting in high Al contents, 22.26 ± 12.7 μg/g in the hippocampus and 640 ± 50 μg/g in the cerebellum respectively. Both showed the increase of oxidative products in the brain tissues. However, much lower Al concentrations in the hippocampus (751.0 ± 225.8 ng/g) and the cerebellum (144.8 ± 36.2 ng/g) could also induce the lipid peroxidation in the brain of the neonatal rat in our study. Therefore, it is quite possible to induce lipid per-oxidation of brain tissues in the neonatal animals even a slightly but significant increase of Al.

### Effects of Al on the activities of anti-oxidative enzymes in the hippocampus, diencephalon, cerebellum, and brain stem

The present study’s analyses comfirmed that high Al could induce oxidative stress and cause lipid peroxidation in the hippocampus, diencephalon, cerebellum, and brain stem. In these tissues, the initiation of antioxidative enzymes to oppose the oxidative stress was expected. Study findings revealed that the activities of SOD decreased with increasing lipid peroxidation in the hippocampus, cerebellum, and brain stem tissues of high Al animals. This was consistent with several other studies which identified significantly decreased SOD activities in the hippocampal region [28,35], cerebellum [33-35], and brain stem [34,35] in high Al animals. However, none of these four studies have measured the final Al concentration of brain tissues, although all had Al-overload through various routes, i.e. drinking water, intra-peritoneal, or oral gavage.

In our present study, the HA group displayed increased catalase activity in the diencephalon. The study of Nehru and Bhalla, however, described decreased catalase activity in the hypothalamus (part of the diencephalon) of high Al-treated rats [30]. The discrepancy of catalase activities between our study and this study might be due to the Al levels in the diencephalon. Unfortunately, no data of Al content was reported in Nehru and Bhalla’ report. Nevertheless, it is believed that the regulation of catalase gene transcription is responded to modification of cellular redox levels. Spiegelman’s study had reviewed that the role of PGC1 transcription coactivators are induced when cells are given ROS. PGC1 includes genes encoding proteins like SOD, catalase, and GPx [36]. Activated OxyR transcription factor switches on several genes encoding antioxidant functions, such as catalase, reported in Dample’s study [37].

In the present study, GPx activity was significantly higher in the cerebellum, hippocampus, and brain stem in the HA group than in the other two groups. Similar results were reported by Esparza et al [32]. On the other hand, opposing results were shown in the hippocampus and brain stem following Al treatment [28,35]. Again, these studies also displayed with no data of the final Al concentration in brain tissue, even with Al-overload by drinking water or intraperitoneally injection.

### Aluminum causes oxidative stress in neonatal brain through production of ROS free radicals

**Figure 3:**
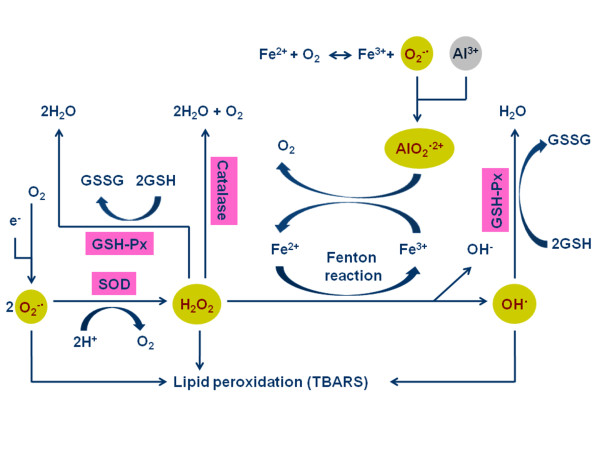
**Diagrammatic representation of the relation among the aluminum (gray), reactive oxygen species (yellow), anti-oxidative enzymes (pink) and lipid peroxidation.** TBARS = thiobarbituric acid reactive substances; SOD = superoxidase dismutase; GPx = glutathione peroxidase. (It is adapted from the research findings of Exley, 2004; Halliwell and Gutteridge, 2007).

The present study identified that Al induced production of the ROS free radical H_2_O_2,_ which participated in oxidative stress in neonatal brain. Figure 3 proposed a theoretical diagram of the relationship between aluminum, ROS, anti-oxidative enzymes, and lipid peroxidation. It is adapted from the research findings of Exley, Halliwell, and Gutteridge [4,38], which suggested Al involved in the iron Fenton reaction in the brain. In this reaction, Al^3+^ (gray), Fe^2+^, and unpaired electrons mediate the production of ROS such as O_2_^**˙-**^, H_2_O_2_, OH^**˙**^, OH^**-**^, and AlO_2_^**˙-**^ (yellow). Superoxide dismutase activity indicated the production of O_2_^**˙-**^, catalase and GPx activities confirmed the production of H_2_O_2_ and OH^**˙**^ (anti-oxidative enzymes; pink). Negatively charged phospholipids of brain cell membranes, which contain polyunsaturated fatty acids, are easily attacked by ROS [4,39,40]. The present study used TBARS to represent peroxidative damage to phospholipids on cell membranes caused by Al. Results of significantly modified oxidative enzymes by Al deposition in neonatal brain tissues confirmed high H_2_O_2_ production in high Al brain. The increased H_2_O_2_ might induce peroxidization in a higher proportion of phospholipids to increase TBARS values. In the present work, the correlation analyses also confirmed the theory. This suggested that oxidative stress caused by high Al content is greater than the protection provided by the anti-oxidizing system; subsequently leading to high possibility of oxidative damage to brain tissue. Esparza et al reported that Al concentration in the brain tissue increased with increasing Al intake, but not in a dose-dependent manner. Oxidative damage occurred in specific brain areas of adult rats [32].

## Conclusions

The results of the present study demonstrate that excess Al accumulates in specific areas of the brain, including the hippocampus, diencephalon, cerebellum, and brain stem in neonatal rats following intraperitoneal injection of high levels of Al. High Al significantly decreased the activities of SOD, but increased the activities of GPx and TBARS level in these specific brain regions. High Al accumulation might induce H_2_O_2_ production. The intraperitoneal administration of 35 mg Al/kg body wt/d, therefore, increased oxidative stress in four specific brain areas in neonatal rats. These findings suggest a possible molecular mechanism for Al-induced oxidative damage in hippocampal and other brain cells. Further investigation of this mechanism, including behavioral analyses, is warranted.

## Abbreviations

Al: aluminum; LA: low Al group; HA: high Al group; ROS: reactive oxygen species; TBARS: thiobarbituric acid reactive substances; SOD: superoxidase dismutase; GPx: glutathione peroxidase.

## Competing interests

The authors declare that they have no competing interests.

## Authors’ contributions

CYY designed the experimental protocol, performed the experiments and drafted the manuscript. GSWH contributed to the study concept, research design, data interpretation and manuscript revision. YJL contributed to the study concept, research design, data interpretation and manuscript revision. All authors read and approved the final version of the manuscript.
